# Persistent Epithelial Defect After Bullae Rupture With Netarsudil

**DOI:** 10.7759/cureus.106819

**Published:** 2026-04-10

**Authors:** Tarannum Mansoori

**Affiliations:** 1 Ophthalmology, Prime Retina Eye Care Center, Hyderabad, IND

**Keywords:** corneal bullae, glaucoma, netarsudil eye drop, persistent epithelial defect, reticular epithelial edema

## Abstract

A 61-year-old male patient presented with reticular epithelial edema (REE) and bullae in the inferonasal quadrant of the right eye (RE) one month after initiation of topical netarsudil 0.02%. The findings were confirmed on anterior segment optical coherence tomography (AS-OCT). Following discontinuation of the medication, the epithelial edema resolved over a period of six weeks.

Nine months later, the patient re-presented with redness, increased lacrimation, and blurred vision in the RE, occurring two weeks after re-initiation of topical netarsudil 0.02%. Slit-lamp examination revealed REE with a few bullae and an epithelial defect in the inferonasal paracentral cornea, corresponding to the location of the previous lesion. Corneal sensations were absent over and in the surrounding area of the lesion. AS-OCT confirmed the presence of epithelial edema and bullae.

Netarsudil was promptly discontinued. The patient was managed with topical lubricants, and a bandage contact lens was applied. Due to a persistent epithelial defect, he subsequently underwent amniotic membrane transplantation combined with lateral tarsorrhaphy. Four weeks later, the epithelial defect had healed, leaving a residual nebular corneal opacity. He later underwent implantation of an Ahmed glaucoma valve (AGV) for intraocular pressure (IOP) control.

Ophthalmologists should be cognizant of the potential corneal adverse effects associated with netarsudil, including REE and bullae formation. In cases of persistent epithelial defects, underlying predisposing factors should be carefully evaluated and managed appropriately.

## Introduction

Netarsudil 0.02% ophthalmic solution is a Rho-associated protein kinase (ROCK) inhibitor and norepinephrine transporter inhibitor indicated for the reduction of intraocular pressure (IOP) in patients with open-angle glaucoma and ocular hypertension. It lowers IOP primarily by increasing aqueous humor outflow through the conventional trabecular pathway via ROCK inhibition. It also reduces episcleral venous pressure and, to a lesser extent, decreases aqueous humor production through inhibition of the norepinephrine transporter.

The most frequently reported adverse event associated with netarsudil is conjunctival hyperemia (approximately 53%), attributed to vasodilation. Less common adverse effects include subconjunctival hemorrhage (approximately 10-15%) and corneal verticillata (approximately 15-25%), as reported in the Rho Kinase Elevated IOP Treatment Trial (ROCKET)-1, ROCKET-2, and ROCKET-4 trials [1-3]. Rare adverse events (5-10%) include increased lacrimation, eyelid erythema, blurred vision, corneal staining, and reduced visual acuity. Isolated case reports have additionally described corneal reticular epithelial edema (REE) and bullous epithelial keratopathy following netarsudil use [4-9].

We report a case of corneal REE, anterior uveitis, and persistent epithelial defect developing after initiation of netarsudil therapy for uncontrolled IOP.

## Case presentation

A 61-year-old male patient with a known history of uncontrolled type 2 diabetes mellitus was referred to the glaucoma clinic for management of elevated IOP in the right eye (RE). His past ocular history was significant for uncomplicated cataract surgery 10 years earlier and pars plana vitrectomy with epiretinal membrane peeling, endolaser photocoagulation, and silicone oil tamponade in the RE, for vitreous hemorrhage, one year prior. He also reported poor vision in the left eye (LE) for the preceding 10 years. He gave history of proliferative diabetic retinopathy in the LE, leading to a long-standing tractional retinal detachment and hypotony.

On examination, best-corrected spectacle visual acuity (BCSVA) was 20/200 in the RE and no light perception in the LE. Anterior segment evaluation of the RE revealed a clear cornea with fine endothelial pigmentation, a deep and quiet anterior chamber, and a posterior chamber intraocular lens in the capsular bag. The IOP measured 34 mmHg in the RE despite maximal tolerated medical therapy, including topical travoprost 0.004% once daily, a fixed combination of dorzolamide/timolol twice daily, and brimonidine tartrate 0.1% three times daily. The IOP in the LE was 4 mmHg.

Gonioscopy of the RE demonstrated open angles up to the scleral spur. Fundus examination revealed an attached retina, a medium-sized optic disc with a cup-to-disc ratio of 0.9, and a bipolar notch. Central corneal thickness in the RE measured 485 µm.

Oral acetazolamide 250 mg twice daily was added to his existing regimen. One week later, the IOP decreased to 19 mmHg. Ahmed glaucoma valve (AGV) implantation was advised for the RE; however, the patient declined surgical intervention. Consequently, topical netarsudil 0.02% once daily was initiated. At one-week follow-up, the IOP was16 mmHg on maximal antiglaucoma medications (AGMs).

One month later, the patient presented with complaints of redness, lacrimation, and hazy vision in the RE. BCSVA had declined to counting fingers at 3 m. Slit-lamp examination revealed diffuse conjunctival congestion with an inferior subconjunctival hemorrhage. The cornea demonstrated REE with bullae in the inferonasal paracentral region and fine keratic precipitates on the endothelium. The anterior chamber showed 1+ cells and flare. IOP measured 10 mmHg on the prescribed AGMs.

**Figure 1:**
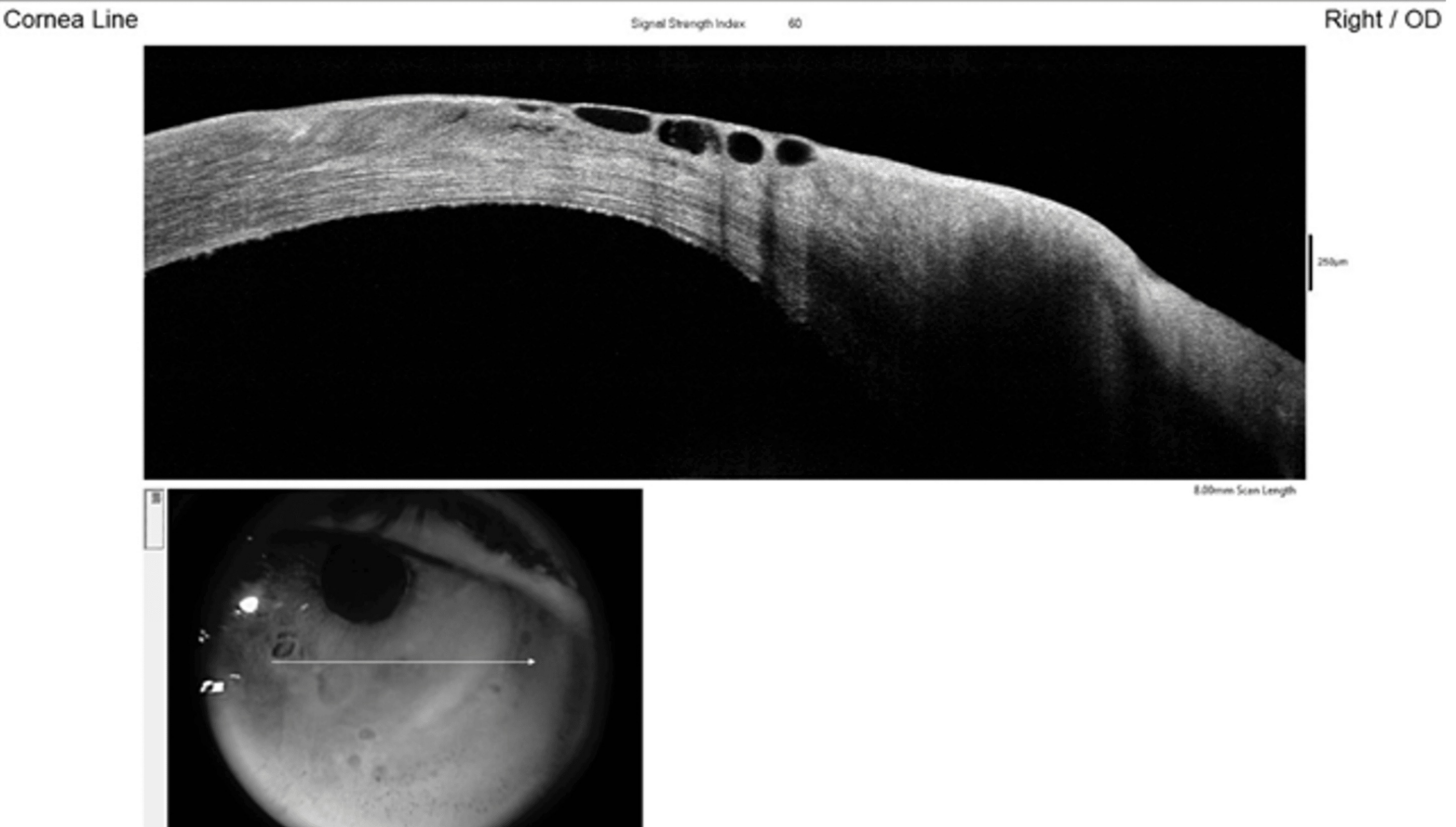
AS-OCT of the cornea demonstrating epithelial edema with multiple bullae and no underlying stromal edema Arrow indicates the imaging plane of the corneal line scan. AS-OCT: Anterior segment optical coherence tomography

Anterior segment optical coherence tomography (AS-OCT) line scan of the cornea demonstrated epithelial edema with bullae, without associated stromal edema (Figure 1).

**Figure 2:**
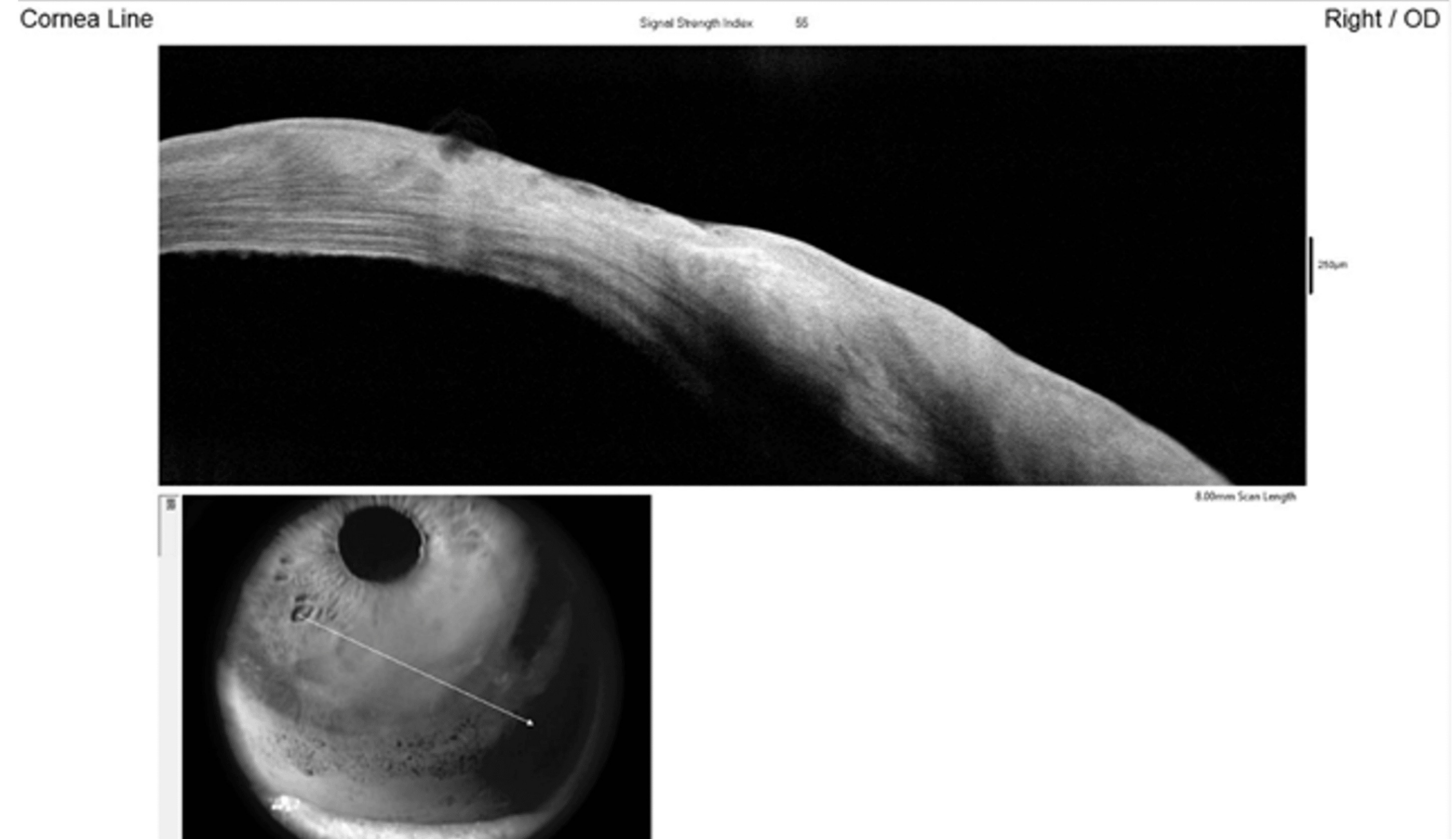
AS-OCT corneal line scan showing resolution of epithelial edema and bullae following discontinuation of netarsudil Arrow indicates the imaging plane of the corneal line scan. AS-OCT: Anterior segment optical coherence tomography

Netarsudil was discontinued, and topical Loteprednol etabonate 0.5% was initiated four times daily. The frequency of oral acetazolamide 250 mg was increased to three times daily. At the six-week follow-up, the patient was asymptomatic, with complete resolution of corneal epithelial edema and bullae. Corneal sensation was assessed using a cotton wisp stimulus and was found to be absent. AS-OCT confirmed resolution of epithelial changes (Figure 2).

The patient was subsequently lost to follow-up.

Nine months later, the patient presented with redness, increased lacrimation, and blurred vision in the RE, which had developed two weeks after re-initiation of topical netarsudil 0.02%, prescribed elsewhere for elevated IOP. Best-corrected visual acuity was counting fingers close to the face.

**Figure 3:**
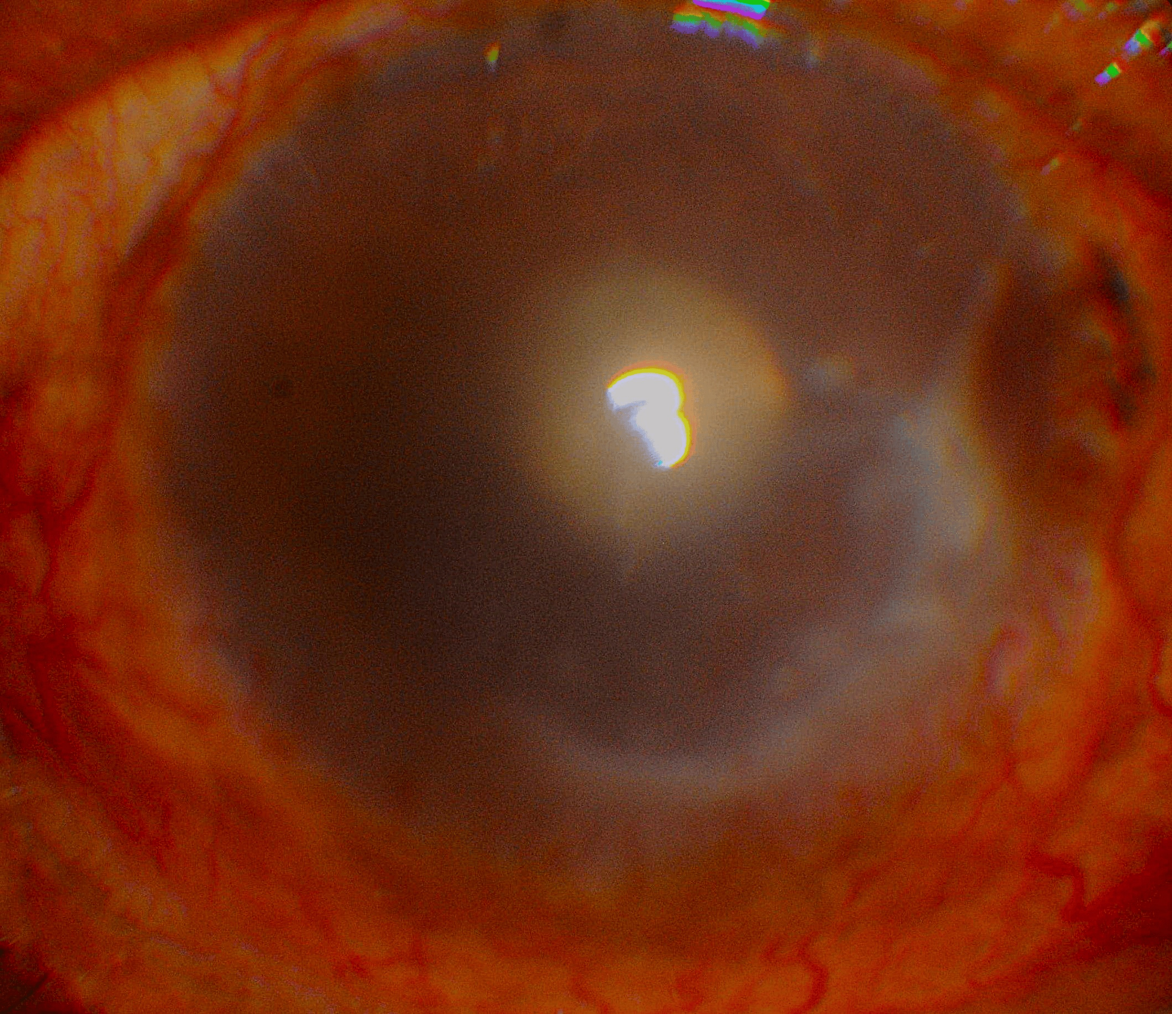
Slit-lamp photograph revealing REE, bullae, and an epithelial defect in the inferonasal paracentral cornea REE: Reticular epithelial edema

Slit-lamp examination revealed circumcorneal ciliary congestion with prominent episcleral vessels. The cornea demonstrated a 5 × 3 mm epithelial defect in the inferonasal paracentral region (Figure 3), corresponding to the site of prior corneal edema. 

**Figure 4:**
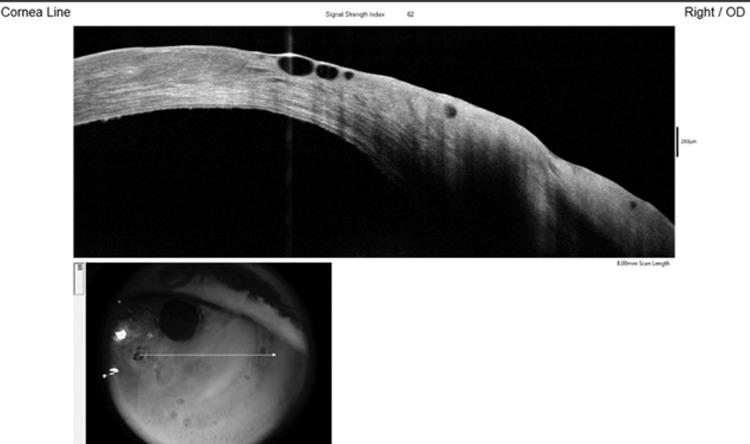
line scan of the cornea showing epithelial edema with a few bullae and the epithelial defect in the inferonasal quadrant Arrow indicates the imaging plane of the corneal line scan. AS-OCT: Anterior segment optical coherence tomography

AS-OCT demonstrated epithelial edema with a few bullae and a corresponding epithelial defect (Figure 4).

Netarsudil was discontinued, and the remaining AGMs were continued. Corneal scrapings for smear and culture were negative for bacterial and fungal organisms. The patient was managed with topical moxifloxacin 0.5% four times daily, carboxymethylcellulose 0.5% six times daily, and loteprednol etabonate 0.5% four times daily. A bandage contact lens was applied.

Despite maximal medical therapy, there was no clinical improvement. Two weeks later, the patient underwent amniotic membrane transplantation combined with lateral tarsorrhaphy.

Postoperatively, topical moxifloxacin 0.5% four times daily and carboxymethylcellulose 0.5% six times daily were continued along with the existing AGMs. In addition, topical 50% autologous serum was prescribed four times daily.

At four-week follow-up, BCSVA improved to counting fingers at ½ m. The corneal epithelial defect had healed, leaving a residual nebular opacity. However, the IOP was elevated at 32 mmHg despite maximal tolerated AGMs.

**Figure 5:**
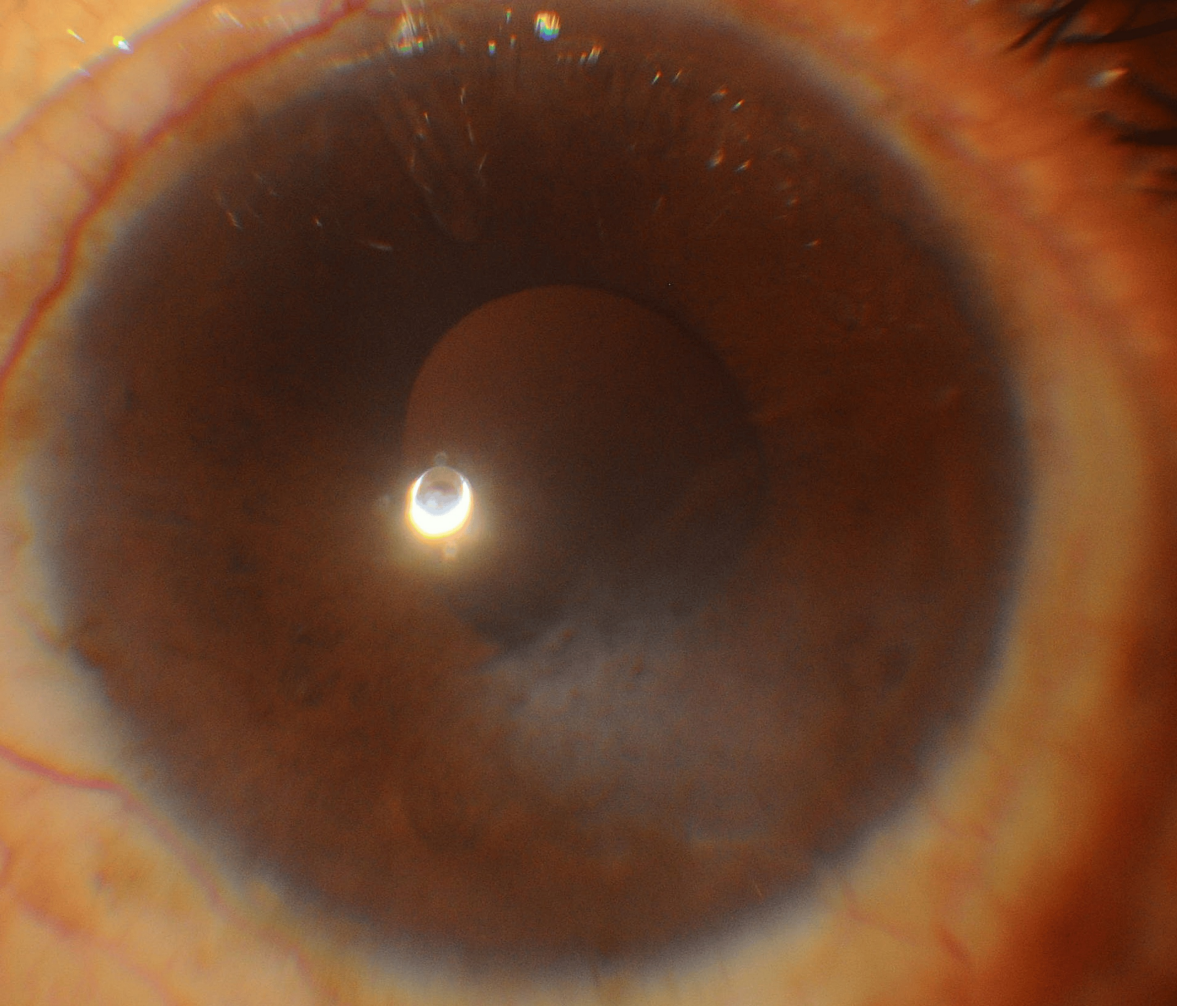
Slit-lamp photograph showing a nebular scar in the inferonasal paracentral cornea; the AGV tube is visible in the anterior chamber at 11 o'clock AGV: Ahmed glaucoma valve

The patient was counseled and subsequently underwent implantation of an AGV (Model FP8, New World Medical, Rancho Cucamonga, USA) for IOP control. One week postoperatively, best-corrected visual acuity remained counting fingers at ½ m. The eye was quiet, and slit-lamp examination revealed a nebular scar in the inferonasal paracentral cornea. No keratic precipitates were noted, the anterior chamber was quiet, and the AGV tube was well positioned within the anterior chamber (Figure 5).

**Figure 6:**
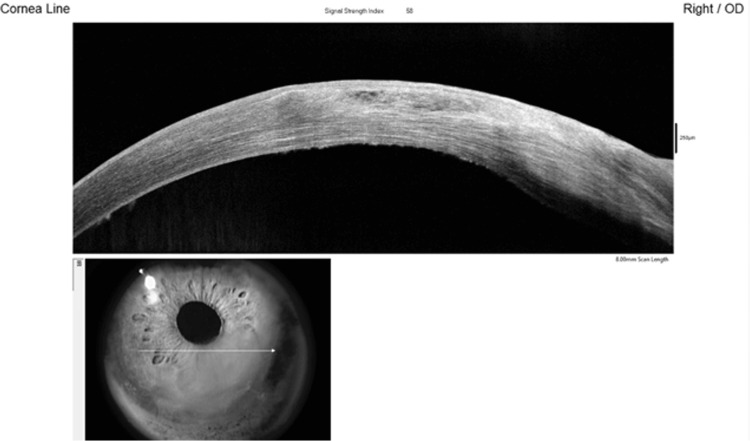
AS-OCT of the cornea demonstrating resolution of epithelial edema and bullae with a healed epithelial defect Arrow indicates the imaging plane of the corneal line scan. AS-OCT: Anterior segment optical coherence tomography

AS-OCT line scan confirmed resolution of the epithelial edema and bullae (Figure 6).

## Discussion

Corneal REE and bullae has been reported in association with ROCK inhibitors, particularly in patients with endothelial cell dysfunction and pre-existing corneal edema [3-9]. The precise pathophysiological mechanism underlying the development of REE and epithelial bullae remains incompletely understood.

One proposed hypothesis suggests that ROCK inhibition enhances endothelial pump function while simultaneously altering tight junction permeability [10]. This may facilitate the translocation of fluid from the corneal stroma toward the epithelium. In eyes with pre-existing stromal edema, redistribution of fluid may preferentially reduce stromal hydration while promoting fluid accumulation within the epithelial layers. As a consequence, stromal edema may appear to improve, whereas epithelial edema and bullae formation become clinically evident. 

A study suggests that the effects of ROCK inhibitors on epithelial tight junctions are reversible following drug discontinuation, which may account for the resolution of epithelial edema observed after cessation of Netarsudil [11]. Alterations in epithelial intercellular junctions may impede the normal percolation of fluid between epithelial cells, thereby limiting subsequent clearance through evaporation and contributing to the persistence of epithelial edema. In addition, ROCK inhibitors are known to influence epithelial wound healing by modulating cellular migration, proliferation, maturation, and adhesion. Such effects on epithelial dynamics may further contribute to the development or persistence of epithelial defects in susceptible individuals.

In a retrospective case series, six episodes of REE in five eyes of five patients treated with topical netarsudil was reported. Four of the five patients had pre-existing corneal edema in the affected eye prior to initiation of therapy. The fifth patient had a history of anterior uveitis and a glaucoma drainage device in the anterior chamber, both of which may have predisposed to endothelial compromise and subsequent corneal edema. In four of the six episodes, prompt clinical improvement of REE was observed within five weeks of discontinuing netarsudil [4].

Another case series involving four patients further highlighted the association between netarsudil and REE. In all cases, cystic epithelial bullae were intact, with no associated epithelial defects. A consistent risk factor identified across these patients was the presence of pre-existing corneal edema, suggesting that endothelial dysfunction may predispose to the development of this distinctive epithelial cystic pattern [6].

Persistent corneal epithelial defects may occur in the setting of neurotrophic keratitis or toxic keratopathy secondary to topical medications. These conditions disrupt epithelial cell migration and impair hemidesmosomal adhesion between the epithelium and the underlying basement membrane. Impaired corneal innervation, an important contributor to neurotrophic keratopathy, may result from diabetes mellitus, severe dry eye disease, current or prior herpetic keratitis, topical anesthetic abuse, or traumatic and postoperative nerve injury.

Our case illustrates the clinical course and a strong temporal association between topical netarsudil therapy and the development of REE, corneal bullae, and anterior uveitis, all of which resolved upon discontinuation of the drug. Notably, the patient developed epithelial bullae at the same inferonasal corneal location following re-initiation of netarsudil, accompanied by a persistent epithelial defect.

We hypothesize that the persistent epithelial defect may have resulted from rupture of the epithelial bullae, potentially occurring during unsupervised use of netarsudil during the second course of therapy. A predisposing factor in this patient appears to be underlying neurotrophic keratopathy secondary to uncontrolled diabetes mellitus. The diagnosis of neurotrophic keratopathy was established based on clinical history and examination findings, as the patient had no prior history of corneal edema and did not report ocular pain.

## Conclusions

In conclusion, ophthalmologists should be aware of the potential adverse effects of topical netarsudil, including corneal REE and bullae formation, particularly in patients presenting with blurred vision or ocular discomfort after initiation of therapy. Patients should be counseled to promptly report new visual changes or ocular symptoms to allow timely evaluation and intervention. In patients who do not report ocular pain, careful assessment of corneal sensation is recommended. In cases of persistent epithelial defects, underlying predisposing factors,such as, neurotrophic keratopathy, prior ocular surgery, or endothelial dysfunction, should be identified and managed appropriately.
